# Better Educated Physicians

**Published:** 1916-11

**Authors:** 


### Better Educated Physicians.

It is stated that more than half the physicians of New York find it difficult to live on the money received from the practice of their profession. This does not indicate so much that the profession is overcrowded as that too many physicians are flocking to the centers of population. They are allured to the cities because they know that should they succeed there the rewards are greater than in the sparsely settled sections. People flock to centers for treatment instead of seeking it in isolated places. So long as New York or any other city holds out the inducements of more profitable returns, just so long will the report come that a large percentage of its physicians are not making decent livings.

This is not true of the medical profession alone. More lawyers and other professional men are half starved in New York than elsewhere. While the city favors the few in all the professions and vocations, and the world hears of the great successes of those few, it never learns of the miseries of the many whose lives are worse than failures. But since people, and physicians among them, will be allured to cities in great number, what must they do to keep from meeting the fate of the many. The ethics of the profession prevent them from advertising, from seeking to build themselves up as those engaged in business may do, from letting their abilities be heralded in the press; so they must rely solely upon either their preparation or upon some influential personal acquaintanceship. Even good social ties will not suffice if preparation be lacking. It follows that physicians who contemplate going to the cities should be certain that they stand out conspicuously above the average members of the profession in professional training and ability. In short, the city physician of the future must be better educated as a physician than the 50 per cent who fail.

What is true of the city is measurably true of all sections of the country. There is hardly room for the great number who secure license to practice medicine. Improved sanitary conditions are improving the general health of the public. It is now regarded as little short of a disgrace for people to have some diseases that a few years ago were regarded as unpreventable. The world is learning better how to care for the human body and prevent the inroads of disease. As the care of the body becomes better understood, fewer physicians will be needed to serve a given population. But as the population increases, about the same proportion will naturally seek to become physicians. It follows, then, that, to succeed, the standards of admission to the profession must gradually be raised, and that he who would have success beyond his fellow workers, must seek a higher degree of efficiency than they.

In short, the successful physician of the future must study, and must study hard, not only for admission to the profession, but to retain his position when once admitted. The time has passed when a diploma from some weak medical college signifies that the physician need not further pursue his studies.

